# Family and Community Nurses as a Resource for the Inclusion of Youths with Type 1 Diabetes at School

**DOI:** 10.3390/jpm13060981

**Published:** 2023-06-11

**Authors:** Maria Brentari, Roberto Franceschi, Jessica Longhini, Evelina Maines, Enza Mozzillo, Marco Marigliano, Cinzia Vivori

**Affiliations:** 1 Community Nurse, Azienda Provinciale per i Servizi Sanitari, APSS, 38123 Trento, Italy; maria.brentari@apss.tn.it; 2 Pediatric Diabetology Unit, Pediatric Department, S.Chiara General Hospital of Trento, APSS, 38122 Trento, Italy; roberto.franceschi@apss.tn.it (R.F.); evelina.maines@apss.tn.it (E.M.); 3 Department of Diagnostics and Public Health, University of Verona, 37129 Verona, Italy; jessica.longhini@univr.it; 4 Department of Translational Medical Science, Section of Pediatrics, Regional Center of Pediatric Diabetes, Federico II University of Naples, 80138 Naples, Italy; 5 Department of Surgery, Dentistry, Pediatrics and Gynecology, Section of Pediatric Diabetes and Metabolism, University and Azienda Ospedaliera Universitaria Integrata of Verona, 37126 Verona, Italy; marco.marigliano@univr.it; 6 Hygiene and Public Prevention Department, Azienda Provinciale per i Servizi Sanitari, APSS, 38123 Trento, Italy; cinzia.vivori@apss.tn.it

**Keywords:** type 1 diabetes, school, family and community nurse

## Abstract

School nurses can facilitate the inclusion of students with type 1 diabetes (T1D) at school; this model has been widespread in some countries but not in Italy, which is due to the insufficient number of school nurses that are able to provide medical attention at all times. The National Recovery and Resilience Plan (PNRR) devised a series of aids and support for the reorganization of the Italian National Health System (NHS) through the creation of community houses in addition to family and community nurses (FCNs), who will operate in these structures to promote the integration of the various professional figures and community services. In this study, starting with the needs and suggestions of teachers (No. 79) and parents (No. 48) collected using a survey, we developed a new model for the inclusion of students at school where FCNs who have experience in pediatric T1D have the role of an educator, coordinator, and facilitator’ they cannot be on site and available all the time during school hours, so they must make many efforts to improve the school staff’s knowledge, intervene to offer training when requested, and solve new emerging problems.

## 1. Introduction

In May 2020, the European Commission approved the National Recovery and Resilience Plan (PNRR), which devised a series of aids and support for the reorganization of the Italian National Health System (NHS) through the creation of community houses. Recent experiences related to the COVID-19 pandemic have raised awareness both in citizens and in institutions to the importance of nurses, not only in emergency care management but as an educator in health and good practices [1]. Family and community nurses (FCNs) will operate in these structures to promote the integration of various professional figures and community services; the nurses take care of people, follow them within the familial context, and help them to deal with disease or chronic disability [1]. The purpose of these initiatives is to provide an effective response to the specific conditions of the patients, guaranteeing equal opportunities for access to quality assistance [2]. In Europe, the role of FCNs varies with respect to the contexts and changes according to the needs of the population, particularly focusing on frail conditions, maternal and child support, or the prevention and management of chronic diseases [3]. They have specialized postgraduate training and primarily work in community centers, patients’ homes, and with families [3]. FCNs ensure a continuous and coordinated integration between hospitals and primary care services, improving the care continuity of the healthcare system. The use of FCNs is progressing in different contexts, as has been recently summarized in a review [1]; their use ranges from the promotion of healthy habits to educational interventions and supporting secondary healthcare, especially for diabetes mellitus and chronic heart failure patients [1]. The extent and impact of FCNs for patients with diabetes has only been evaluated in patients with type 2 diabetes (T2D) who have developed chronic complications that interferes with daily living and can request assistance from family members or nurses; FCNs provide assistance to these patients for wound care and try to augment patient education and insulin treatment adherence [4,5].

In this scenario, we considered the possible role of using FCNs to promote the inclusion of children with type 1 diabetes (T1D) at school. An evaluation of the needs and management of patients with T1D in a school setting, along with parents’ and teachers’ perceptions, has been performed in 2011 on an Italian cohort through the ALBA project [3]. The study showed that among the school staff that had a student with T1D in their class, only 40.4% received specific training to help children with T1D [6]. At school, insulin was either self-administered or the children had help from parents; a nurse was rarely present (3.6%) and a teacher was rarely available for insulin administration (2.9%). The glucagon question was another issue, as a small percentage of teachers (14.9%) stated they would use glucagon directly in an emergency [6]. Similar results were replicated in studies in Spain: only 45.2% of teachers declared that they have received specialized information on the illness, and they considered the material and human resources as insufficient, calling for the presence of school nurses [7,8]. Parents often reported a lack of teacher knowledge, access to diabetes tools, freedom to perform diabetes self-care, nutritional information, full-time school nurses, and communication between parents and school personnel [6,9].

Previous studies have reported that school nurses can facilitate the application of the individual health plan (IHP) and daily management of T1D [10] through different interventions, which are included in national and international guidelines [11,12]; the nurses can support school glucose monitoring, insulin injection, the prevention and treatment of hypoglycemia, the treatment of high blood glucose level, provide education and counseling for the school staff, and support and arrange meetings with school counselors as needed [13,14]. These interventions have been reported to provide improved adherence to self-care, better glucose control, and better psychological outcomes (reduced distress, improved quality of life, and satisfaction) [10,15,16] if the school nurse has less than five students with T1D [11]. This model has been widespread in some provinces but not in others in the same country, which is due to the insufficient number of school nurses that are able to provide medical attention at all times [17]. Only 35.3% of all public schools in the United States employ full-time nurses and, over time, the number of school nurses employed by districts has decreased by 14% [18]. These school nurses provide acute and chronic care, including care coordination, assessments, health education and promotion, and emergency preparedness [18]. They provide direct services to students, communicate with caregivers via phone, and collaborate with local health departments, but almost three-fourths of schools utilize the school staff to help in their work [18]. In Italy, the Regional Health Service can provide a nurse to be present at specified times, but only if no school staff member is available because of the scarcity of assistant nurses [6]. Therefore, the role of the school staff in assisting children and adolescents with T1D is a particular interesting area of research.

The aims of this study were: (i) to investigate parents’ and teachers’ needs in our province and to collect suggestions about the possible role of the FCNs to facilitate and protect the school integration of children with T1D; and (ii) to develop a new model for inclusion at school, based on FCNs, who could serve as a sustainable alternative to the school nurse.

## 2. Subjects and Methods

### 2.1. Setting, Study Design, and Sample

The Autonomous Province of Trento (North of Italy) has a territory that is 100% mountainous; the population density is 87/km^2^, and there are 215 primary schools for 25.290 students. Most of the schools are in small villages, and the number of children with T1D at each school number at most 1 or 2. The figure of the school nurse is not anticipated, while a pool of nurses has been recently trained to be FCNs in the community houses.

In the period of May–June 2022, a descriptive observational study was carried out through a survey to evaluate the needs and suggestions parents and school staff regarding the inclusion of students with T1D at school and the implementation of a school inclusion model involving FCNs.

An online survey was proposed to all 85 parents (members) of the ADGT (Youth Diabetes Association of Trentino), one for each family. In the same period, 208 teachers and school collaborators from pre-primary, primary, and compulsory secondary education, who had at least one child/adolescent with T1D at school and had attended a 2-day course for managing students with T1D, were asked to participate in the study. Family pediatricians and pediatric diabetologists were interviewed as professional figures, involved in the patients’ management, and helped to develop the model.

### 2.2. Ethical Considerations

This study has followed the guidelines and ethical principles of the Declaration of Helsinki. Participants were informed of the objective of the survey, their participation was voluntary, and the completion of the online survey was considered an agreement to participate on the part of parents and school staff. During the online survey, no personal identifying information was collected, and this study complies with the provisions of the General Data Protection Regulation (EU-GDPR-2016) [19].

### 2.3. Parents and School Staff’s Survey

Two questionnaires were specifically designed for the investigation of parents’ and school staff’s needs by the diabetology staff and community nurses based on the ones designed by Carral-San Lauretano et al. [20], Pinelli et al. [6], and Amillategui et al. [21].

The two questionnaires, the one consisting of 18 items for the parents and the other consisting of 23 items for the school staff, were built with the same structure to investigate: (i) sociodemographic characteristics; (ii) individual health plan (IHP) implementation at school; (iii) external support; and (iv) school staff needs or parent needs and suggestions.

(i) Socio-demographic characteristics: Age, sex, working status, child’s age, and nationality. The working status was classified according to the Italian National Institute of Statistics criteria, which is entirely cross-linkable with the International Standard Classification of Occupations (as we previously reported [22]) and was grouped into two levels: low (unoccupied, unskilled, and semi-skilled workers, manual workers, and craftsmen) and high (legislators, senior officials and managers, professionals, technicians, associate professionals, sales workers, small business and farm owners, administrators, and higher executives).
(ii) IHP implementation was evaluated in terms of daily practices at school by using closed (yes/no) and multiple choice questions. A 5-point Likert scale, with the response sets ranging from 1 = I totally disagree to 5 = I strongly agree, was used to explore the glucose levels check, insulin administration, students’ accessibility and participation in school activities, food and dietary management, school staff’s confidence in recognizing and treating hypoglycemia and hyperglycemia, and participation in meetings to increase knowledge of diabetes.
(iii) External support: The questions for parents explored the support provided by the school, healthcare services, and patient association; the questions for the school staff explored the support offered by colleagues, healthcare services, and patient associations. A 5-point Likert scale questionnaire was used.
(iv) The parents’ and school staff’s needs: Using multiple choice questions, we asked about situations in which it was difficult to manage diabetes. Suggestions to improve the model were collected through open-ended as well as 5-point Likert scale questions.

### 2.4. Statistical Analysis

The statistics were analyzed using GraphPad Prism version 8.0.2 (GraphPad, San Diego, CA, USA). Open-ended questions were analyzed through “The qualitative content analysis process” [23] and were translated into quantitative variables. The last ones were tested for statistical normality. The data were expressed as the means ± SD for variables with normal distribution and as medians (interquartile range) for non-normally distributed variables. Differences between groups of continuous variables were analyzed using Student’s t-test for paired samples for the variables with normal distribution or using Wilcoxon signed rank sum test for variables with non-normal distribution. A chi-square test with Fisher’s test was used to evaluate the differences in the categorical data. *p* values < 0.05 were considered significant.

## 3. Results

In total, 48 (56%) parents and 79 (38%) teachers and school collaborators completed the online survey. Of the parents, 81.2% were female, 85.4% were Italian, 67% had a high working status; their child/adolescent had a mean age (standard deviation, SD) of 11.48 (±3.68) years, and all of them were using continuous glucose monitoring (CGM) systems (Table S1). 

Among the school staff, 74% were teachers, 91% were female, and 45.6% were older than 50 years. School staff had 19.7 (mean, SD ± 12.5) years of experience in pre-primary and primary education (54.4%) and in compulsory secondary education (45.5%). Most of the students were 6–13 years old (Table S2). Of the teachers, 66% only monitored one student with T1D; Of the teachers, 76% affirmed that they were aware of the existence of IHP, and 70% had participated in a meeting at the school with healthcare professionals (Table S3).

**Figure 1:**
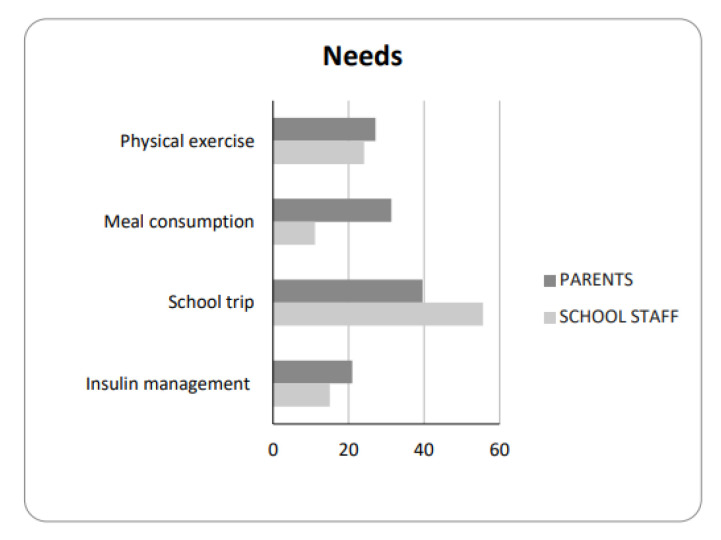
Parents’ and school staff’s needs.

Parents’ survey: Overall, from the parents’ point of view, an inclusive approach at school has emerged, as reported in answers 1 to 9: a high proportion of students had lunch at school (75%) and 92% attended all activities (Table S4). Glycemic control was performed in class, in the garden, or in the gym (85%), which was accomplished by the students themselves (77%), or by teachers (19%). Insulin with pen or pump was given by students (67%) or by teachers in 8% of the cases. As a related result, 81% of parents continued their job. Parents considered the relationships with teachers to be very important (73%), along with the child’s acceptance of the disease (73%). Parents felt supported by school staff, healthcare professionals, and patients’ associations, as reported in the three questions ranging from 13 to 15. Parents’ needs were in the area of school trips (40%) in terms of the management of hypoglycemia, hyperglycemia, and insulin injection; meal consumption (31%), which was recognized as a difficulty that was related to how much food the child can eat, along with difficulties in organizing the administration of insulin at school and establishing the right time of food initiation after taking insulin; physical exercise (PE) (27%) for the frequency of glucose monitoring and hypoglycemia prevention; and insulin management (21%), in terms of who injected the insulin and who determined the dosage decision (Figure 1).

**Figure 2:**
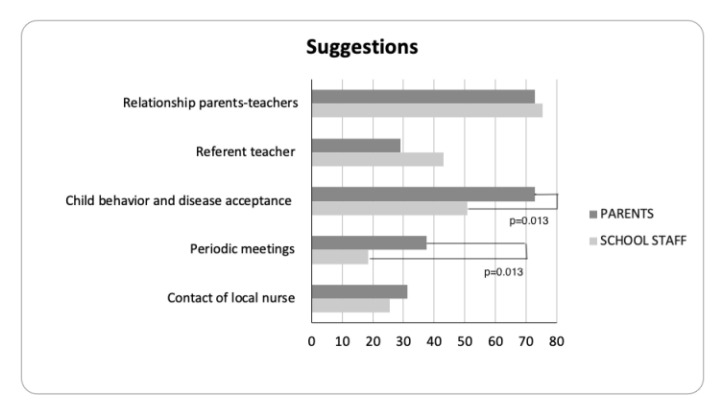
Parents’ and school staff suggestions. Chi-square test with Fisher’s test has been used to evaluate differences in categorical data. *p* values < 0.05 were considered significant.

The parents suggested putting more effort into improving the relationship with teachers (73%), identifying a referent teacher in each school (29%), planning periodic meetings (37%), and working on child disease-related acceptance and behaviors at school (73%). Moreover, they considered community nurses as a figure that could improve T1D management at school (31%) (Figure 2).

School staff’s survey: From questions 1 to 17, there emerged a collaborative approach by the school staff in terms of glucose checking using mostly CGM systems. Students administered insulin on their own with a pen or pump in 48% and 43% of cases, respectively, and it was administered by teachers using the same methods at rates of 21% and 22%, respectively; however, the teachers were not as confident in performing this. School staff referred to feeling quite confident with glucose control (61%), but less so with respect to glucose management in the case of hypoglycemia (40%), PE (25%), and school trip (25%) management. They considered the relationships with parents (63%) and colleagues to be very important (44%), along with the child’s behavior and acceptance of the disease (42%). They felt supported within the school and by healthcare services, and they thought that patient associations played a facilitating role in school integration. The school staff’s needs were in the area of school trips (55.6%), physical exercise (PE) (24%), insulin (15%), and meal consumption (11%) management (Figure 1).

They suggested improving relationships with the parents (75.4%), identifying a referent teacher in each school (43%), organizing periodic meetings (18%), working on child behavior and disease acceptance at school (51%), and they considered community nurses as a figure that could improve T1D management at school (25%) (Figure 2).

When comparing the parents’ and school staff’s needs, no significant differences emerged; while comparing the suggestions from the two groups, parents were more prone to suggest periodic meetings among the different stakeholders (*p* = 0.013) and to work on child behavior and disease acceptance (*p* = 0.013).

### The Possible Role of the FCNs in the Inclusion of Students at School

The proposal of a new model for the inclusion of students at school came from meetings between FCNs trained in T1D, family pediatricians, and pediatric diabetologists, which considered patients’ and school staff’s needs and suggestions and included these points:

(1) Following teachers’ suggestions, we promoted the project called “Good practices in T1D at school”. When a child develops T1D, a referent teacher in each school collaborates with colleagues from other schools that have had similar experiences to improve the knowledge, strategies, and possible solutions according to a “peer”-based approach;
(2) The diabetology staff prepares the IHP and FCNs who have experience in pediatric T1D and organizes a meeting with the school staff and parents. Following the school staff’s and parents’ suggestions, after the first period of student inclusion at school, the FCNs stay in contact with the school, with at least 2–3 monthly teleconsultations with the aim of providing more knowledge on T1D to intercept emerging problems and reinforce good practices;
(3) As suggested by the parents and school staff, the psychologist of the diabetology team contacts the school psychologist to evaluate the child’s behavior and acceptance of the disease at school. At the same time, the dietician of the diabetology team contacts the dietician of the company that dispenses food at school.

## 4. Discussion

The goal of this study was to improve our current model of inclusion for students with T1D at school, starting with the analysis of the parents and school staff needs and analysis of the disease perception, which is conducted while including the figure of FCNs as educators, coordinators, and facilitators.

T1D must be managed 24 h a day, 7 days a week, and it requires continuous care, even when a child attends school or extracurricular activities [24]. Management of T1D is imperative in school settings as children and adolescents spend up to 8 h or longer each weekday at school [9]; positive diabetes management in this context is associated with better metabolic control and a higher quality of life [25]. The challenge is to achieve a balance between insulin doses and nutritional intake and PE. 

Children are educated in the self-management of the disease, but at the age of elementary and middle school, they require the assistance of adults, which is often provided by the child’s parents, school nurses, teachers, or other school personnel [15]. Adolescents may require help eating or drinking to treat hypoglycemia and have to be supervised until the low values return to a normal range; sometimes, they need a reminder to self-administer insulin bolus at lunch. The school nurse can provide a link between the student, the school, and home, with the nurses being available to supervise and assist with routine disease management; however, in our country, there are few school nurses that are currently available, and they are only available in some of the provinces.

Our previous model of inclusion of students with T1D considered that members of the diabetology team, school staff, and students’ parents have to collaborate and coordinate with each other in accordance with the students’ IHP. The diabetology staff usually provide a basic training to all the school personnel to ensure the management of hypoglycemia, hyperglycemia, and, in the case of an emergency, one or more school staff members receives in-depth training on insulin and/or technology management at their request.

To improve this model, we started by analyzing the school staff and parents’ needs with a survey; a good level of knowledge in T1D and an adequate level of teacher training have been reported. This premise was different from other studies in which teachers referred to having inadequate training [17], probably because in our province, the protocol for the inclusion of children with T1D at school provides collegial and/or individual meetings between diabetology staff and school staff. Secondly, an inclusive approach at school has emerged, as a high percentage of students with T1D could participate in different school activities. The school staff was confident about glucose checks, which is probably thanks to the widespread use of CGM systems and because 20% of them can administer insulin at school, via pen or pump, with parents’ advice. We have interpreted this data considering that during the 2-day course for managing students with T1D, teachers and school collaborators have the opportunity to participate in practical sessions on glucose control and insulin administration. In a previous survey in Italy, only 2.9% of teachers took responsibility for the treatment, and this was the result of private agreements between the families and the schools [6]. However, also in our study, needs in four areas were reported by the parents and school staff: meal consumption, PE, school trips, and insulin management; these were similarly reported in previous studies [6]. Some areas of improvement were reported from both parties: the importance of a good relationship between the parents and school staff, the establishment of a referent teacher, and the possibility to contact a local nurse. The last two figures were not available at the time of the survey, and to the best of our knowledge, the first area of improvement has not been previously described in literature; the second was probably cited in view of school nurse experiences that are available in the other provinces of Italy [6]. 

The parents also suggested more attention on periodic meetings after starting school to intercept new emerging problems, and they asked for an evaluation of child acceptance of the disease and of their relationships with peers.

On the basis of these premises, we explored the possibility of including a resource that was only recently available in our province: the FCNs. In our country, they work in community centers and in patients’ homes, improving the care continuity between hospitals and primary care services; however, thinking about their involvement with respect to the inclusion of students with T1D at school, they could not be on-site and available all the time during school hours. Therefore, we developed a model in which the FCNs act as a glue between the different figures, i.e., as a coordinator and facilitator, and intervene in person to offer training to the school staff, solve new emerging problems, and reinforce good practices at school [24]. Moreover, for this task, the use of telemedicine could shorten distances in some mountainous areas of our country, as experienced during the SARS-CoV2 pandemic [26,27].

FCNs should be familiar with current pediatric diabetes information and management strategies [9], and according to these requirements, our community nurses attended full-day educational programs. As for school nurses, FCNs frequently need updated information due to the consistent advancement in the technology applied to T1D [28]; in particular they have up-to-date CGM techniques and insulin pump knowledge.

We included in the model a project called “Good practice at school”, which was created at the school staff’s suggestion because they recognized the importance of sharing knowledge about diabetes in children with other colleagues to understand and be able to communicate with the child and family [24]. Teachers are trained to educate, not to manage chronic healthcare conditions, and they meet one or two students with T1D in their career; therefore, colleagues from other schools could refer about their experience, scenarios (including hypoglycemia, hyperglycemia, and exercise management), and projects that have involved classmates concerning healthy eating, diabetes, and individual diversity.

However, a good relationship between the family and school staff, even if very important, could be insufficient for improving the child’s acceptance of the disease at school. For children and adolescents, returning to school after a diagnosis of diabetes is not easy; they can feel anger, sadness, and a need for calm, reassurance, and privacy [6]. They are worried about class learning and participation in all school activities along with their relationship with peers; these feelings should not be minimized, as they can imply adverse effects on the students’ cognitive and educational performance at school [6] and on their adherence to insulin treatment and metabolic control [29]. Therefore, in the model, we have included the contact between psychologists of the school and the diabetology team for the purposes of sharing information; concerns with respect to the child’s relationships with peers have also emerged in other studies [6], and psychologists could help teachers learn how to deal with some situations or behavior and how to meet the child’s and classmates’ expectations.

This study presents some limitations: (i) the two questionnaires used were not previously validated, in contrast to the ones previously used in literature; and (ii) we did not consider questions on glucagon storage and administration because intranasal glucagon spray has been available since November 2021 in Italy. In a previous survey in Italy [6], half of parents said that their school did not want to store glucagon, and only a small percentage of teachers (23%) considered their school as being able to manage severe hypoglycemia; additionally, only 14.9% of teachers said that they would use glucagon directly in an emergency [6].

The strengths of this study are as follows: (i) we developed a model starting from actors’ needs and suggestions, which should be tailored on the basis of students’ age, individual level of self-management, and acceptance of the disease according to a precision medicine-based approach [30,31,32]; and (ii) this model is more sustainable in our reality compared with one involving school nurses and complies with the indications of the PNRR.

In conclusion, starting from parents’ and school staff’s needs, we developed a model wherein FCNs have the role of an educator, coordinator, and facilitator for the inclusion of youths with type 1 diabetes at school, which is in compliance with and supported by PNRR aids. FCNs cannot be on site and available all the time during school hours; therefore, they may make many efforts to improve school staff’s knowledge and organize periodic meeting to face new emerging problems, with the aim of allowing students to fully participate in school activities, improving their diabetes control as well as their quality of life.

## Data Availability

All databases generated for this study are included in the article.

## Supplementary Materials

The following supporting information can be downloaded at: https://www.mdpi.com/article/10.3390/jpm13060981/s1, Table S1. Parents’ and child’s socio-demographic characteristics and information on diabetes management (*n* = 48); Table S2. School staff characteristics (*n* = 79); Table S3. Data from school staff survey (*n* = 79); Table S4. Data form parents’ survey results (*n* = 48).

## References

[1] Dellafiore F., Caruso R., Cossu M., Russo S., Baroni I., Barello S., Vangone I., Acampora M., Conte G., Magon A. (2022). The State of the Evidence about the Family and Community Nurse: A Systematic Review. Int. J. Environ. Res. Public Health.

[2] Piano Nazionale della Cronicità. https://www.salute.gov.it/imgs/C_17_pubblicazioni_2584_allegato.pdf.

[3] Conti A., Albanesi B., Busca E., Martini L., Costa C., Campagna S. (2021). L’infermiere di famiglia e comunità: Panoramica sull’esercizio della professione in Europa [Family and community nursing: Overview of practice in Europe]. Assist. Inferm. Ric..

[4] Davis W., Lewin G., Davis T., Bruce D. (2013). Determinants and costs of community nursing in patients with type 2 diabetes from a community-based observational study: The Fremantle Diabetes Study. Int. J. Nurs. Stud..

[5] Lumbers M. (2021). Osteomyelitis, diabetic foot ulcers and the role of the community nurse. Br. J. Community Nurs..

[6] Pinelli L., Zaffani S., Cappa M., Carboniero V., Cerutti F., Cherubini V., Chiarelli F., Colombini M., La Loggia A., Pisanti P. (2011). The ALBA Project: An Evaluation of Needs, Management, Fears of Italian Young Patients with Type 1 Diabetes in a School Setting and an Evaluation of Parents’ and Teachers’ Perceptions. Pediatr. Diabetes.

[7] Armas Junco L., Fernández-Hawrylak M. (2022). Teachers and Parents’ Perceptions of Care for Students with Type 1 Diabetes Mellitus and Their Needs in the School Setting. Children.

[8] Wilt L. (2022). The Role of School Nurse Presence in Parent and Student Perceptions of Helpfulness, Safety, and Satisfaction With Type 1 Diabetes Care. J. Sch. Nurs..

[9] Kise S.S., Hopkins A., Burke S. (2017). Improving School Experiences for Adolescents With Type 1 Diabetes. J. Sch. Health.

[10] Dai H., Chen Q., Huang H., Wu K., Yang X. (2022). The Role of Nurses in Taking Care of Children With Type 1 Diabetes. Altern. Health Med..

[11] West E., Holmes J. (2014). The Role of the School Nurse in the Management of Diabetes: Assessing a Position Statement. Br. J. Sch. Nurs..

[12] Wilt L., Jameson B., Maughan E.D. (2021). School Nursing Evidence-Based Clinical Practice Guideline: Students with Type I Diabetes. https://learn.nasn.org/courses/33787.

[13] Stefanowicz A., Stefanowicz J. (2018). The Role of a School Nurse in the Care of a Child with Diabetes Mellitus Type 1-the Perspectives of Patients and Their Parents: Literature Review. Slov. J. Public Health.

[14] Wang Y.-L., Volker D.L. (2013). Caring for Students With Type 1 Diabetes: School Nurses’ Experiences. J. Sch. Nurs..

[15] Peery A.I., Engelke M.K., Swanson M.S. (2012). Parent and Teacher Perceptions of the Impact of School Nurse Interventions on Children’s Self-Management of Diabetes. J. Sch. Nurs..

[16] Wilt L. (2021). The Relationships Among School Nurse to Student Ratios, Self-Efficacy, and Glycemic Control in Adolescents With Type 1 Diabetes. J. Sch. Nurs..

[17] Luque-Vara T., Fernández-Gómez E., Linares-Manrique M., Navarro-Prado S., Sánchez-Ojeda M.A., Enrique-Mirón C. (2021). Attitudes and Perceptions of School Teachers in Melilla Regarding the Care Provided to Students with Type 1 Diabetes. Children.

[18] Willgerodt M.A., Brock D.M., Maughan E.M. (2018). Public School Nursing Practice in the United States. J. Sch. Nurs..

[19] (2016). Data Protection Regulation (EU) (GDPR) (2016) Regulation (EU) 2016/679 of the European Parliament and of the Council of 27 April 2016 on the protection of natural persons with regard to the processing of personal data and on the free movement of such data, and repealing Directive 95/46/EC (General Data Protection Regulation). Off. J. Eur. Union.

[20] Carral San Laureano F., Gutiérrez Manzanedo J.V., Moreno Vides P., de Castro Maqueda G., Fernández Santos J.R., Ponce González J.G., Ortega A. (2018). Actitudes y percepción del profesorado de centros educativos públicos sobre la atención a lumnus con diabetes tipo 1. Endocrinol. Diabetes Y Nutr..

[21] Amillategui B., Calle J.R., Alvarez M.A., Cardiel M.A., Barrio R. (2007). Identifying the Special Needs of Children with Type 1 Diabetes in the School Setting. An Overview of Parents’ Perceptions: Original Article. Diabet. Med..

[22] Girardi M., Assalone C., Maines E., Genovese A., Naselli A., Nai Fovino L., Soffiati M., Franceschi R. (2022). Disease Characteristics and Psychiatric Comorbidities in Adolescents with Anorexia Nervosa Hospitalized During COVID-19 Pandemic. Front. Biosci. (Sch. Ed).

[23] Elo S., Kyngäs H. (2008). The Qualitative Content Analysis Process. J. Adv. Nurs..

[24] American Diabetes Association Safe at School, Helping the Student with Diabetes Succeed: A Guide for School Personell. https://diabetes.org/sites/default/files/2022-11/School-guide-final-11-16-22.pdf.

[25] Wagner J., Heapy A., James A., Abbott G. (2006). Glycemic control, quality of life, and school experiences among students with diabetes. J. Pediatr. Psychol..

[26] Tornese G., Schiaffini R., Mozzillo E., Franceschi R., Frongia A.P., Scaramuzza A. (2021). The Effect of the COVID-19 Pandemic on Telemedicine in Pediatric Diabetes Centers in Italy: Results from a Longitudinal Survey. Diabetes Res. Clin. Pract..

[27] Tornese G., Schiaffini R., Mozzillo E., Franceschi R., Frongia A., Scaramuzza A., on behalf of HCL Expert Pathway Pediatric Group, the Diabetes Study Group of the Italian Society for Pediatric Endocrinology (2021). Telemedicine in the Time of the COVID-19 Pandemic: Results from the First Survey among Italian Pediatric Diabetes Centers. Healthcare.

[28] Fisher K. (2006). School nurses’ perceptions of self-efficacy in providing diabetes care. J. Sch. Nurs..

[29] Rabbone I., Minuto N., Toni S., Lombardo F., Iafusco D., Marigliano M., Schiaffini R., Maltoni G., Frongia A.P., Scardapane M. (2018). Insulin pump breakdow and infusion set failure in Italian children with typ 1 diabetes: A 1-year prospective observational study with suggestions to minimize clinical impact. Diabetes Obes. Metab..

[30] Franceschi R. (2022). Precision Medicine in Diabetes, Current Research and Future Perspectives. J. Pers. Med..

[31] Franceschi R., Canale M., Piras E.M., Galvagni L., Vivori C., Cauvin V., Soffiati M., Maines E. (2022). Influence of Parental Health Locus of Control on Behavior, Self-Management and Metabolic Control, in Pediatric Patients with Type 1 Diabetes. J. Pers. Med..

[32] Maltoni G., Franceschi R., Di Natale V., Al-Qaisi R., Greco V., Bertorelli R., De Sanctis V., Quattrone A., Mantovani V., Cauvin V. (2022). Next Generation Sequencing Analysis of MODY-X Patients: A Case Report Series. J. Pers. Med..

