# Supplementary material for: Family and Community Nurses as a Resource for the Inclusion of Youths with Type 1 Diabetes at School
Source: J Pers Med. 2023 Jun 11;13(6):981. doi: 10.3390/jpm13060981 (PMC10302974; doi:10.3390/jpm13060981)
Supplement: Supplementary file 1 [file jpm-13-00981-s001.zip › jpm-2380249-supplementary.pdf]

Table S1. Parents' and child's socio-demographic characteristics and information on diabetes management (N=48)

**Parents' characteristics**	N (%)
**Age (years),** Mean ± SD	45.31±5.64
**Gender: female/male**	39 (81.2) / 9 (18.8)
**Parent educational level**	
Middle school graduate	1 (2.1)
High school graduate	30 (62.5)
University studies	17 (35.4)
**Working status**	
Low (unoccupied/housewife/unskilled, semi-skilled, manual workers and craftsmen)	16 (33)
High	32 (67)
**Nationality**	
Italian	41 (85.4)
Morocco, Romania, Albania, Macedonia	7 (14.6)
**Children's age (years),** Mean ± SD	11.48±3.68
**Diabetes duration (years),** Mean ± SD	5.83±4.11
**School level**	
Elementary school	17 (35.4)
Middle school	31 (64.6)
**Insulin regimen: MDI / CSII**	21 (44) / 27 (56)
**CGM systems use, Yes**	48 (100)

Legend. CGM: continuous glucose monitoring; CSII: continuous subcutaneous insulin infusion; MDI: multiple daily injections; N, Number; SD, Standard Deviation.

Table S2. School staff characteristics (N=79).

School staff characteristics	N (%)
**Age**	
20-30 years	3 (3.7)
30-40 years	10 (12.7)
40-50 years	30 (38)
> 50 years	36 (45.6)
**Gender: female/male**	72 (91.1) / 7 (8.9)
**School education level**	
Secondary school diploma	47 (59.5)
University studies	32 (40.5)
**Employment in the school**	
Teacher	59 (74)
School collaborator	16 (20.5)
Professional educator	4 (5.5)
**Work experience (years),** Mean ± SD	19.7±12.5
**Place of work**	
Infant school	6 (7.6)
Elementary school	37 (46.8)
Middle school	36 (45.5)
**Teacher with N of students with T1D**	
1 student	52 (66)
> 1 student	27 (34)
**Student's age**	
3-5 years	3 (3.8%)
6-10 years	43 (54.4)
11-13 years	31 (39.2)
14-18 years	2 (2.6%)
**Students' insulin regimen: MDI / CSII**	40 (51%) / 39 (49%)

Legend. CSII: continuous subcutaneous insulin infusion; MDI: multiple daily injections; N, Number T1D: type 1 diabetes.

Table S3. Data from school staff survey (n=79).

Question	N (%)
IHP implementation at school	
1. Do you feel confident about glucose level checking? Mean ± SD*	3.22±1.29
2. How do you check glucose levels?	
Only sensor	37 (47)
Sensor + capillary	33 (42)
Only capillary check	9 (11)
3. Where is the glucose check performed?	
In the classroom, in the courtyard or in the gym	59 (75)
In a separate place	20 (25)
4. Do you feel confident about managing glucose levels? Mean ± SD*	2.83±1.17
5. Is lunch eaten at school? Yes	57 (72)
6. How is insulin administered?	
CSII	37 (47)
MDI	42 (53)
7. Who mainly administer insulin with the pen? (N= 42)	
Student	20 (48)
Parent	13 (31)
Teacher with parents' support	9 (21)
8. Who mainly administers insulin with the pump? (N=37)	
Student	16 (43)
Parent	12 (32)
Teacher with parents' support	8 (22)
Community Nurse	1 (3)
9. Do you feel confident in administering insulin with the pump? Mean ± SD*	2.67± 1.40
10. Are you aware of the existence of the Individual Health Plan (IHP) for students with diabetes?	
No	12 (15)
Yes	60 (76)
I don't know what it is	7 (9)
11. Have you ever attended the 2-day course on diabetes at school, in the last 5 years? Yes	57 (74)
12. Have you ever participated in a meeting in the school on diabetes management, with health care professionals? Yes	23 (30)
13. Which figures participated in the meeting? (N=25)	
Pediatric diabetologist, school staff, family, of which:	22 (88)
Plus a nurse	2
Plus a general practitioner	1
14. In what situations do you feel confident in managing diabetes at school? (Maximum 3 answers)	
Glucose check	48 (61)
Hypoglycemia management	32 (40)
meal management	22 (28)
PE management	20 (25)
Trip	20 (25)
15.What do you think makes the difference in the management of children with diabetes? (Maximum 3 answers)	
Relationship with parents	50 (63)
with other teachers and school collaborators	35 (44)
with health care professionals	10 (13)
Child's behavior	33 (42)
Child's acceptance of the disease	33 (42)
Age at diabetes onset	12 (15)
Diabetes duration	6 (8)

Relationship with classmates	10 (13)
Number of children in the class	14 (18)
School policies on the management of students with T1D	10 (13)
Linguistic barrier	10 (13)
16. Have you participated in events organized by the patients' association, or did you consult their website (Youth Diabetes Association of Trentino)?	
No	59 (75)
Yes	20 (25)
17. Do you know if your students with T1D have participated in school camps organized to improve diabetes self-management?	
No	31 (39)
Yes	19 (24)
I don't know	29 (37)
External support	
20. Do you feel supported in the management of the child with T1D within the school? Mean ± SD*	3.35±0.88
21. How much do you feel supported by the health care services? Mean ± SD*	3.58±0.80
22. Do you think patient associations play a facilitating role in school integration? Mean ± SD*	3.67±1.21
School staff's needs	
18. In what situations do you have difficulties in managing diabetes?	
Trip	44 (55.6)
PE	19 (24)
Insulin injection	12 (15)
None	12 (15)
Meal at school	14 (11)
Birthday parties at school	5 (4)
Hypoglycemia	6 (7)
19. In what situations did you need to contact health professionals (diabetologists, nurses, general practitioner)?	
None	60 (76)
Glycemic variability	7 (9)
Problems in relationships with parents	6 (8)
Insulin dosing	4 (5)
Meal	2 (2)
Suggestions	
23. What could help you to deal with these situations?	
Better relationship with parents	59 (75,4)
Working on child behavior and disease acceptance	40 (51)
Referent teacher in each school	34 (43)
Co-presence with a nurse	20 (25)
Periodic meetings with families and healthcare professionals' Possibility to	14 (18)
contact pediatric diabetologist	4 (5)
Training courses on diabetes at school	9 (11)
Patient association support	6 (8)

Legend. CGM: continuous glucose monitoring; CSII: continuous subcutaneous insulin infusion; IHP: individual healthcare plan; MDI: multiple daily injections; PE: physical exercise, T1D: type 1 diabetes. * Normal range 1-5.

Table S4. Data form parents' survey results (n=48)

Question	N (%)
IHP implementation at school	
1. Did you have to quit your job because of your son/daughter's diabetes? Yes	9 (19)
2. Does your child have lunch at school?	
Yes	36 (75)
No	8 (17)
No due to diabetes	4 (8)
3. Where is glycemic control mostly done?	
In class, garden, gym	41 (85)
In a separate place	7 (15)
4. Who mainly performs glycemic control at school?	
Student	37 (77)
Parent	2 (4)
Teacher	9 (19)
5. Who mainly administers insulin with a pen or pump at school?	
Student	32 (67)
Parent	12 (25)
Teacher	4 (8)
Health care professional	0 (0)
6. Has your child had severe hypoglycemia (loss of consciousness and/or seizures) at school in the past year? Yes	2 (4)
7. Please identify some school activity where the student has been excluded due to T1D	
None	44 (92)
School Trip	3 (6)
Physical activity	1 (2)
8. Has your child participated in diabetes school camps? Yes	29 (60)
9. What makes the difference in school management? (Maximum 3)	
Relationship with teachers	35 (73)
Child's behavior and state of acceptance of the disease	35 (73)
Age at the onset of diabetes	18 (37)
Relationship with classmates	15 (31)
School policies on the management of children with diabetes	12 (25)
Diabetes duration	6 (12)
Relationship with health care professionals	6 (12)
Parents' needs	
10. In what situations did you have difficulty managing diabetes at school?	
School trip	19 (40)
Meal consumption in the canteen	15 (31)
PE	13 (27)
Insulin management	10 (21)
Birthday party	8 (17)
Management of snacks at school	7 (14)
Glucose checks	7 (14)
Management of hypoglycemia	7 (14)
11. In what situations did you need to contact health professionals (diabetologists, nurses, pediatrician / family doctor) (select a maximum of 3 answers)	
Insulin dosage	18 (37)
Meal management at school	12 (25)
Relationships with teachers for child management	10 (21)
PE	8 (17)
Hyperglycemia	7 (14)

Significant changes in glucose levels during school hours	7 (14)
Hypoglycemia	4 (8)
School trip	4 (8)
Administration of insulin	3 (6)
12. For what reasons did you consult the pediatrician for the management of diabetes at school?	
None	31 (65)
Teachers' education	8 (17)
Meal	4 (8)
Infections	2 (4)
Dose adjustment	1 (2)
Hyperglycemia	1 (2)
Document preparation	1 (2)
External support	
13. How much do you feel supported in managing your child with T1D at school? Mean ± SD*	3.44±1.12
14. In case of need, do you feel supported by the health care services in diabetes management at school? Mean ± SD*	3.96±1.18
15. Do patients' associations play a facilitating role in school integration? Mean ± SD*	3.93±1.19
**Suggestions**	
16. How could the inclusion of children at school be improved? (Maximum three answers)	
Good relationship with teachers	35 (73)
Working on child behavior and acceptance of the disease	35 (73)
Referent teacher in each school	14 (29)
Periodic meetings with family Pediatrician, Diabetologist, Nurse	18 (37)
Possibility to contact community nurse	15 (31)
Possibility to contact diabetologists	9 (19)
17. Do you think that other figures such as the community nurse, could integrate and improve the relationship between school and family? Mean ± SD*	4.35± 0.96
18. Could a good relationship with family / school / health professionals facilitate diabetes management? Mean ± SD*	4.87 ±0.49

Legend. N, Number; PE: physical exercise; T1D: type 1 diabetes. * Normal range 1-5.
